# Serum CTRP9 and high-molecular weight adiponectin are associated with ischemic stroke

**DOI:** 10.1186/s12883-022-02967-w

**Published:** 2022-11-15

**Authors:** Yan-Qing Zhang, Yan-Wei Zhang, Jian-Li Dai, Chen Li, Wen-Qing Wang, Hai-Feng Zhang, Wayne Bond Lau, Xiao-Ming Wang, Xiao-Gang Liu, Rong Li

**Affiliations:** 1 Department of Anesthesiology, School of Anesthesiology, The First Hospital, Shanxi Medical University, Taiyuan, Shanxi China; 2 Department of Cardiology, The First Hospital, Shanxi Medical University, Taiyuan, Shanxi China; 3 Department of Geriatrics, Xijing Hospital, Air Force Military Medical University, Shaanxi Xi’an, China; 4 Department of Hematology, Tangdu Hospital, Air Force Military Medical University, Xi’an, Shaanxi China; 5 Department of Teaching and Experiment Center, Air Force Military Medical University, Xi’an, Shaanxi China; 6 Department of Emergency Medicine, Thomas Jefferson University, Philadelphia, PA USA; 7 The Key Laboratory of Biomedical Information Engineering of Ministry of Education, School of Life Science and Technology, Xi’an Jiaotong University, Shaanxi Xi’an, China

**Keywords:** Ischemic stroke, CTRP9, Adiponectin

## Abstract

**Background:**

C1q/TNF-related protein 9 (CTRP9) and adiponectin (APN) have beneficial metabolic regulatory and vasoprotective effects. This study explored alteration of CTRP9 and APN multimers during onset of ischemic stroke and development, to provide novel clinical and experimental basis for recognition and prevention of ischemic stroke.

**Methods:**

There were 269 patients with ischemic stroke and 182 control subjects included in this study. Serum levels of CTRP9 and APN multimers in different disease stages were measured.

**Results:**

Serum CTRP9, total APN (tAPN), and high-molecular weight (HMW) APN decreased gradually in stage I (acute stage, within 72 h of onset) of ischemic stroke and increased during stage III (11th day to one month) and stage IV (1 month after), compared to control. In the non-hyperlipidemia group, serum CTRP9, tAPN, and HMW were decreased in ischemic stroke patients compared to control (*P* < 0.05). Serum CTRP9 is closely related to serum tAPN and HMW (*r* = 0.992, 0.991). Serum CTRP9 are protective against ischemic stroke (OR = 0.400, 95% CI 0.197–0.810, *P* < 0.05).

**Conclusions:**

Lower serum CTRP9, tAPN, LMW, and HMW are significantly associated with increased ischemic stroke risk in non-hyperlipidemia subjects. CTRP9, tAPN, and HMW isoforms may be valuable clinical indicators for patients with ischemic stroke.

**Supplementary Information:**

The online version contains supplementary material available at 10.1186/s12883-022-02967-w.

## Background

Cerebral ischemic stroke refers to localized ischemic softening or necrosis of brain parenchyma caused by cerebral circulation disorders, secondary ischemia, and hypoxic pathological changes [1]. Stroke, the second leading cause of death in the world only after ischemic heart disease, is the leading cause of disability in adults [2]. Cerebral ischemic stroke accounts for 87% of all strokes [3].

It has been well-established that adipose tissue has active secretory functions [4]. It secretes various adipokines involved in many pathophysiological processes, such as inflammation, energy metabolism, apoptosis, and aging [4–6]. Among the adipokines, leptin, adiponectin (APN), and C1q/TNF-related proteins (CTRPs) all play active roles in energy metabolism regulation, vasomotion modulation, platelet activation, and inflammation reaction [7–9]. Serum APN exists in three major forms: trimer (low-molecular weight, LMW), hexamer (middle-molecular weight, MMW), and multimer (high-molecular weight, HMW) which contains a bouquet of 12–18 (possibly more) monomers [10]. Different APN isoforms, known to exert tissue-specific biological function and to activate distinct signaling pathways, do not interconvert in circulation [11–14]. Of the three APN isoforms, the HMW multimer represents the majority of total adiponectin (tAPN) and is the major bioactive isoform [15]. Substantial evidence supports CTRP9 and APN as beneficial molecules against obesity-related cardiovascular diseases and glycolipid disorders [7, 16]. However, the potential implications of them in clinical settings have not been well documented. In this study, we determined the levels of serum CTRP9 and different APN multimers in patients of different stroke stages and non-stroke subjects, to define the association of CTRP9/APN multimer levels, as well as dynamic changes after onset, with ischemic stroke, therefore to explore their potentials as risk factors and/or biomarkers. Accomplishment of this study would help to identify high-risk individuals of stroke onset and developing worse outcomes. The association between APN and CTRP9 with stroke may also provide new insights into the pathology of stroke onset and following staging.

## Methods

### Study sample

There were 269 patients with ischemic stroke recruited from departments of neurology, geriatrics, and emergency medicine of Xijing Hospital (Xi’an, China) from October 2016 to November 2017. All patients met the diagnostic criteria of the “Guidelines for the Prevention of Stroke in Patients with Stroke and Transient Ischemic Attack” (American Heart Association and American Stroke Association, 2014) [1]. Infarction was confirmed by CT or MRI. All patients were classified into four subgroups based upon established ischemic stroke staging criteria [17]. There were 57 cases of hyperacute or acute stage (Stage I, < 72 h of infarction), 54 cases of subacute stage (Stage II, 4–10 days after onset), 38 cases of chronic stage (Stage III, 11–30 days after onset), and 115 cases of late chronic stage (Stage IV, > 1 month after onset). Five cases of undetermined onset date were excluded from the stage subgroup analyses.

Study subjects without ischemic stroke (*n* = 182) were recruited from the departments of neurology and geriatrics of Xijing hospital concurrently. CT or MRI verified all control individuals were absent of ischemic or infarct lesions.

To avoid potential confounding factors and control heterogeneity of the study sample, we excluded subjects with any of the following conditions: (1) presence or history of cerebral hemorrhage without pre-existing ischemic stroke; (2) history of ischemic encephalopathy of other causes; (3) previous or current diagnosis of coronary heart disease or coronary syndrome.

The study was approved by the Institutional Review Board of Xijing Hospital (Approval No.: XJ20160443). All study participants gave written consent for study participation.

### Clinical and laboratory examination

About 4ml of peripheral blood was collected immediately after confirmation of ischemic stroke in the emergency department, or at 6:00 a.m. the next morning of admission. The blood was placed into EDTA tube for 2 h at room temperature, and centrifuged for 15 min at 3000 r/min. The serum supernatant was stored in a labelled cryopreservation tube at –80 °C freezer until laboratory assays. Hospital records documenting clinical course and laboratory assays were reviewed after patient discharge. We did not have information of height and weight due to difficulty in obtaining accurate values for commonly bedridden patients. Hyperlipidemia was diagnosed according to 2016 Chinese guideline for the management of dyslipidemia in adults [18]. Specifically, patients with total cholesterol ≥ 6.2, low-density lipoprotein (LDL) cholesterol ≥ 4.1, high-density lipoprotein (HDL) cholesterol < 1.0, or blood triglyeride ≥ 2.3 were diagnosed as hyperlipidemia. The transcranial Doppler ultrasound and carotid ultrasound were used to determine the presence of intracranial (stenosis) and extracranial (plaque) atherosclerosis.

Serum CTRP9 and APN concentrations were measured by use of double antibody sandwich avidin–biotin peroxdase complex-enzyme-linked immunosorbent assay (ABC-ELISA) kit (Shanghai Xitang, China) per manufacturer’s protocol. Each blood sample was measured twice and the measurements were averaged for subsequent analyses.

### Statistical analysis

Data management and statistical analyses were performed using Excel 2010 and SPSS 19.0. Continuous observations were expressed as mean ± SD. Difference between groups were tested by Student’s t test or *t'* test. The skew-distributed data were subjected to Mann–Whitney U test or Kruskal–Wallis H (K) test. Counting data are conveyed as count (percentage) and subjected to chi-square test. Spearman correlation tested the correlation of quantitative data between groups. Risk factors were determined by use of logistic regression. *P* values less than 0.05 were considered significant.

When comparing the differences of serum APN/CTRP9 between stroke and non-stroke groups, we applied propensity score to control potential between-group imbalance of confounding parameters. Specifically, multivariable logistic regression was used to derive a propensity score for stroke onset of all subjects. When comparing the between-group differences of APN/CTRP9, the inverse probability weighting approach [19] based on the propensity scores was applied for the purpose of adjustment.

## Results

Table 1 reveals the baseline characteristics of the study subjects. There was no significant difference in serum CTRP9 and APN levels between infarct versus control groups (*p* > 0.05). Age, being of male gender, tobacco use, alcohol consumption rate, proportion of family history of cardiovascular and cerebrovascular diseases, systolic blood pressure, diastolic blood pressure, fasting serum glucose, glycosylated hemoglobin, levels of homocysteine, prevalence of diabetes mellitus, hypertension, atrial fibrillation, vitamin B12 deficiency, arrhythmia, stenosis, and occlusion of intracranial and carotid artery, and atherosclerosis were all greater in the ischemic stroke group compared to control (*p* < 0.05) before adjustment was applied. After adjustment for propensity score derived using all foregoing characteristics except APN isoforms and CTRP9, we did not find any characteristics showing significant association with stroke onset. The concentration of total cholesterol, HDL cholesterol, LDL cholesterol, and the ratio of hepatic adipose infiltration were decreased in the ischemic stroke group compared to control (*p* < 0.01; Table 1).

**Table 1:** Comparison of serum CTRP9 and APN levels and baseline characteristics of the study subjects between the cerebral infarction group and control

	**Control (*n* = 182)**	**Cerebral infarction (*n* = 269)**	***P*-value**	
			**Before adjustment**	**After adjustment**
CTRP9 (ng/ml)	0.58 ± 0.58	0.43 ± 0.62	0.066	0.071
tAPN (μg/ml)	13.69 ± 8.50	12.05 ± 8.32	0.089	0.082
HMW (μg/ml)	13.13 ± 8.54	11.26 ± 8.35	0.070	0.066
MMW (ng/ml)	7.27 ± 5.96	7.36 ± 8.46	0.91	0.76
LMW (μg/ml)	0.71 ± 0.25	0.72 ± 0.26	1.00	0.87
Age (year)	50.80 ± 15.37	59.34 ± 15.07	< 0.001	0.091
Male gender (%)	108(59.3)	203(75.5)	< 0.001	0.12
Current smoking (%)	28(15.4)	87(32.3)	< 0.001	0.20
Alcohol consumption (%)	34(18.7)	82(30.5)	< 0.01	0.24
Family history (%)	22(12.1)	77(28.6)	< 0.001	0.87
Systolic BP(mmHg)	127.54 ± 19.22	137.22 ± 24.15	< 0.001	0.08
Diastolic BP(mmHg)	79.04 ± 10.66	81.61 ± 13.56	< 0.001	0.11
Hypertension (%)	66(36.3)	171(63.6)	< 0.001	0.67
Fasting serum glucose(mmol/L)	4.82 ± 0.73	5.17 ± 1.28	< 0.001	0.34
Glycosylated hemoglobin (%)	5.6(0.9)	6.5(2.2)	< 0.001	0.31
Diabetes mellitus (%)	18(9.9)	64(23.8)	< 0.001	0.09
Atrial fibrillation (%)	4(2.2)	18(6.7)	< 0.05	0.17
Arrhythmia (%)	17(9.3)	43(16.0)	< 0.05	0.52
Total cholesterol (mmol/L)	4.32 ± 0.93	3.74 ± 0.98	< 0.001	0.18
Triglyceride (mmol/L)	1.29 ± 0.71	1.30 ± 0.70	0.88	0.76
HDL cholesterol (mmol/L)	1.18 ± 0.30	1.02 ± 0.27	< 0.001	0.25
LDL cholesterol (mmol/L)	2.32 ± 0.63	2.08 ± 0.71	< 0.01	0.41
Hyperlipidemia (%)	67(36.8)	87(32.3)	0.33	0.51
Homocysteine(μmol/L)	11.22 ± 4.65	12.82 ± 4.48	< 0.01	0.16
Vitamin B 12 Deficiency (%)	40(22.0)	107(39.8)	< 0.001	0.19
Low blood folic acid (%)	66(36.3)	120(44.6)	0.08	0.18
Stenosis and occlusion of intracranial and carotid artery (%)	4(2.2)	89(33.1)	< 0.001	0.11
Atherosclerosis (%)	67(36.8)	139(51.7)	< 0.01	0.24
Hepatic adipose infiltration (%)	37(20.3)	30(11.2)	< 0.01	0.13

Continuous data are presented as mean ± SD. Categorical data are presented as count (percentage)

All subjects were assigned to four subgroups: subjects without ischemic stroke and hyperlipidemia (Group I, *n* = 115), ischemic stroke patients without hyperlipidemia (Group II, *n* = 182), control subjects with hyperlipidemia (Group III, *n* = 67) and patients with hyperlipidemia (Group IV, *n* = 87). Serum CTRP9, tAPN, HMW APN and LMW APN were significantly decreased in the hyperlipidemic group compared to the non-hyperlipidemic group (*p* < 0.01, Table 2). The non-hyperlipidemic group exhibited significantly decreased serum levels of CTRP9, tAPN, and HMW APN in Group II compared to Group I (*p* < 0.05, Table 3). Quantitative data and ordinal data between two non-hyperlipidemia subgroups demonstrated positive correlation between serum CTRP9 concentration and serum levels of all APN isoforms (including tAPN, HMW, MMW, and LMW APN), age, HDL, and vitamin B12 (*p* < 0.01), and negative correlation with diastolic blood pressure and serum triglyceride levels (*p* < 0.01, Table 4).

**Table 2:** Comparison of serum CTRP9 and APN levels of hyperlipidemic and non-hyperlipidemic subjects

	**Non-hyperlipidemia (*n* = 297)**	**Hyperlipidemia (*n* = 154)**	***P*-value**
CTRP9(ng/ml)	0.59 ± 0.60	0.39 ± 0.45	< 0.01
tAPN(μg/ml)	14.17 ± 9.39	11.16 ± 6.78	< 0.01
HMW(μg/ml)	13.41 ± 9.19	10.54 ± 6.77	< 0.01
MMW(ng/ml)	7.06 ± 7.50	7.48 ± 5.81	0.54
LMW(μg/ml)	0.76 ± 0.23	0.64 ± 0.22	< 0.001

The measurements are presented as mean ± SD

**Table 3:** Comparison of serum CTRP9 and APN levels of cerebral infarct study subjects versus control, with respect to hyperlipidemia

	**Non- hyperlipidemia**			**Hyperlipidemia**		
	**Group I (*n* = 115)**	**Group II (*n* = 182)**	***P*-value**	**Group III (*n* = 67)**	**Group IV (*n* = 87)**	***P*-value**
CTRP9(ng/ml)	0.66 ± 0.57	0.44 ± 0.62	< 0.05	0.39 ± 0.37	0.38 ± 0.55	NS
tAPN(μg/ml)	15.28 ± 9.23	12.30 ± 8.81	< 0.05	11.33 ± 5.58	11.16 ± 8.29	NS
HMW(μg/ml)	14.17 ± 8.97	11.51 ± 8.93	< 0.05	11.03 ± 5.58	10.42 ± 8.53	NS
MMW(ng/ml)	7.38 ± 6.37	6.90 ± 8.53	NS	6.50 ± 4.47	7.82 ± 7.88	NS
LMW(μg/ml)	0.76 ± 0.20	0.76 ± 0.26	NS	0.59 ± 0.22	0.68 ± 0.22	< 0.05

The measurements are presented as mean ± SD

*NS* Not significant

**Table 4:** Correlation analysis with serum CTRP9 levels in the non-hyperlipidemia group

	***r***_***s***_	***P*-value**
tAPN	0.992	< 0.001
HMW	0.991	< 0.001
MMW	0.179	< 0.01
LMW	0.424	< 0.001
Age	0.251	< 0.001
Systolic BP	-0.050	NS
Diastolic BP	-0.232	< 0.001
Fasting serum glucose	-0.091	NS
Glycosylated hemoglobin	-0.148	NS
Total cholesterol	0.095	NS
Triglyceride	-0.279	< 0.001
HDL cholesterol	0.359	< 0.001
LDL cholesterol	-0.060	NS
Homocysteine	-0.120	NS
Vitamin B12	0.214	< 0.01
Folic acid	0.043	NS

*NS* Not significant

In the non-hyperlipidemic group, thirteen independent variables (age, sex, smoking status, drinking history, family history, hypertension, diabetes mellitus, homocysteine, vitamin B12 and CTRP9, HMW, MMW, LMW) were included in the binary logistic regression for analyzing ischemic stroke risk factors. Age exceeding 60 [OR (95% CI), 3.305 (1.573–6.947), *p* < 0.01], smoking [2.434 (1.074–5.520), *p* < 0.05], hypertension [3.557 (1.742–7.263), *p* < 0.001] and vitamin B12 deficiency [2.653 (1.206–5.835), *p* < 0.05] were independent risk factors to ischemic stroke. High CTRP9 concentration was a protective factor against ischemic stroke [0.400 (0.197—0.810); *p* < 0.05] (Table 5).

**Table 5:** Binary logistic regression analysis with backward selection for cerebral infarct presence in the non-hyperlipidemia group

	**OR (95% CI)**	***P*-value**
Aged over 60	3.305 (1.573, 6.947)	< 0.01
Current smoking status	2.434 (1.074, 5.520)	< 0.05
Hypertension	3.557 (1.742, 7.263)	< 0.001
Vitamin B12 Deficiency	2.653 (1.206, 5.835)	< 0.05
CTRP9	0.400 (0.197, 0.810)	< 0.05

Age, sex, current smoking status, drinking history, family history, hypertension, diabetes mellitus, homocysteine, vitamin B12, and CTRP9, HMW, MMW, LMW were included as independent variables. The classification criteria were 60 years old, homocysteine 15.0 μmol/L, vitamin B12 211 pmol/L, CTRP9 0.5869 ng/ml, HMW 13.3067 μg/ml, MMW 7.0978 ng/ml, LMW 0.7582 μg/ml

The levels of serum CTRP9 and various APN isoforms in patients at different stages of ischemic stroke were compared against control. Serum CTRP9, tAPN, and HMW APN decreased gradually after ischemic stroke onset, which decreases in stage II and stage III were statistically significant (*p* < 0.05), and restored in Stage IV. MMW and LMW APN exhibited significant transient increase in Stage I. (*p* < 0.05, Table S1).

## Discussion

To the best of our knowledge, this is the first study comprehensively investigating the association of serum CTRP9 and APN isoforms with cerebral ischemic stroke onset and stages in a clinical setting. We reported several significant findings. First, the serum concentration of CTRP9, tAPN, and HMW APN significantly decrease in ischemic stroke patients without hyperlipidemia. Elevated CTRP9 level is likely to exert independently protective effect against stroke onset. Second, serum CTRP9, tAPN, and HMW APN gradually decrease initially and eventually restored by the late stage infarct period.

Systematic inflammatory response is actively involved in cerebral infarction development [20]. Cerebral infarction shares common pathological mechanisms with cardiovascular diseases such as coronary heart disease [21]. Clinical trials demonstrated that increased CTRP9 is an independent protective factor for coronary heart disease [22]. To exclusively determine the role of serum CTRP9 and APN in cerebral infarct pathophysiology, we patients of excluded coronary heart disease from this study.

A long list of predisposing factors are associated with ischemic stroke risk, including but not limited to age, hypertension, type 2 diabetes, obesity, hyperlipidemia, and heart diseases, etc. [1, 23]. More than 40% of stroke patients have no such risk condition [24]. Obesity, a well-documented modifiable risk factor, is characterized by derangements of adipose-derived hormones [23, 25]. APN (also known as Acrp30, AdipoQ, GBP-28, and apM1) is the most abundant peptide secreted by adipocytes, and plays a central role in obesity-related diseases [26]. Studies have demonstrated that APN has insulin-sensitizing, anti-atherogenic, and anti-inflammatory effects [27, 28].

Leaving aside consistently reported protection, the relationship between serum APN level and risk of ischemic stroke is inconsistent in clinical studies [23]. Decreased APN level has been associated with increased ischemic stroke risk independently or indirectly [29–31]. In contrast, the Northern Manhattan Study (NOMAS) reported association between lower circulating APN level and decreased ischemic stroke risk [32]. A recent meta-analysis also reported a positive association between serum APN level and ischemic stroke risk [33]. In this study we found significant association of APNs/CTRP9 with stroke in subjects with normal blood lipids, which association was not the case in the whole sample. This implies that the major confounding factor behind the inconsistency may be the heterogeneity across the study samples.

The etiology of the inconsistency across studies may also arise from the coexistence of multiple APN isoforms and their temporal patterns in infarct subtypes and stages, given that different metabolic disorders may underlie the same diagnosis of cerebral ischemic stroke. Total concentrations aside, isoform distribution must be considered in the interpretation of serum APN level [34]. A case–control study demonstrated that serum APN concentration in large artery atherosclerotic (LAA) subtype of ischemic stroke patients significantly decreased in non-stroke subjects compared to non-LAA stroke patients, with no significant difference between non-LAA patients and non-stroke subjects [35]. The observed trend in this study that CTRP9, tAPN, and HMW APN decreased gradually and increased to baseline during ischemic stroke is consistent with evidence suggesting that CTRP9 and HMW unequivocally harbor protective effect within the nervous system [36–38]. CTRP9, a novel adipokine first reported in 2009, expresses predominantly in adipose tissue [39]. As a paralog of APN, CTRP9 shares 51% amino acid sequence with APN [39]. CTRP9 regulates energy metabolism, modulates vasomotion, protects endothelial cells, inhibits platelet activation and pathological vascular remodeling, stabilizes atherosclerotic plaques, and protects the heart [40].

Emerging evidence suggests that CTRP9 exerts neuroprotective effects upon central nervous system diseases [41]. CTRP9 has a high affinity to APN receptor 1 (AdipoR1), which is widely expressed in the central nervous system, especially on neurons [42, 43]. AdipoR1 agonists protect the brain against ischemic stroke and intracerebral hemorrhage via inhibiting neuronal apoptosis [36, 44]. Nevertheless, some (albeit few) researches reported the association of serum CTRP9 level with ischemic stroke risk in humans [45]. The levels of serum CTRP9 and tAPN in patients at early stage (within 3 days) of ischemic stroke were decreased compared to control [45]. Heretofore, no investigation focused on the pattern of APN/CTRP9 response to stages of cerebral ischemic stroke to the best of our knowledge.

Closely related to abnormal adipose metabolism, hyperlipidemia is an important and independent risk factor for ischemic stroke [1, 3]. As expected, tAPN, HMW APN, and CTRP9 levels in the hyperlipidemic group were decreased compared to the non-hyperlipidemic group. To avoid the confounding variable of lipid concentration, a subgroup analysis per blood lipid level was completed in this study. In the non-hyperlipidemic group, the levels of CTRP9, tAPN, and HMW APN were decreased in stroke patients compared to control. This implies that decreased levels of CTRP9 and APN may be risk factors of ischemic stroke independent of hyperlipidemia. Previously we reported that the HMW APN isoform is most closely associated with cardiovascular diseases-related biochemical indicators, total cholesterol, high-density lipoprotein cholesterol, and uric acid, and therefore an independent predictor of cardiovascular diseases [15]. Meanwhile, high serum CTRP9 is an independent protective factor for metabolic syndrome, and is correlate with decreased hyperlipidemia indicators such as cholesterol, triglyceride, and low density lipoprotein [46]. In this study, binary logistic regression in the non-hyperlipidemic group revealed age > 60, smoking, hypertension, and low vitamin B12 were risk factors of ischemic stroke, while increased CTRP9 was a protective factor of cerebral infarction. When CTRP9 was removed from the initial model, high concentration of HMW exerted protective factor for ischemic stroke (details not presented). In the non-hyperlipidemic subgroup, serum CTRP9 were positively correlated with APN (including tAPN, HMW, MMW, and LMW APN), age, HDL, and vitamin B12, and negatively correlated with diastolic blood pressure and triglyceride.

Diabetes was surprisingly not among the risk factors of stroke in this study. One of the possible reasons may rest in the fact that patients with coronary heart disease were excluded from this study. Coronary atherosclerosis is the central pathology of coronary heart diseases. As a component of metabolic syndrome, diabetes has extensive crosstalk with hyperlipidemia and atherosclerosis. Therefore, exclusion of patients with coronary heart diseases would simultaneously exclude diabetes patients with more complications. The remaining patients with “benign diabetes”, a term coined presently to represent potential diabetic subtypes imposing less damage to other organs, may contribute to lack of association between diabetes and stroke risk.

Several limitations exist in the current work. First, this is a cross-sectional study, making it difficult to establish causal inference. Second, the sample size is not very large. Further studies with larger sample size are warranted. Third, the effect of concurrent diseases and current active medications were not considered, which may affect serum CTRP9 and/or APN levels and thereby act as confounding factors. This may also underlie the observed paradox of lower total cholesterol and LDL cholesterol, as well as lower prevalence of hyperlipidemia in patients than in controls, as shown in Table 1.

## Conclusions

In summary, serum CTRP9 was closely correlated to tAPN and HMW APN. Increased serum CTRP9 is an independent protective factor for ischemic stroke. Reduced serum CTRP9, tAPN, and HMW APN may impose increased ischemic stroke risk, and may serve as biomarkers of early ischemic stroke stage. Assays of these molecules’ concentrations may increase precision of diagnosis, subtyping, and staging of cerebral ischemic stroke. Large-scale multiple-center collaborative efforts investigating the effects of CTRP9 and APN upon cardiovascular and cerebrovascular diseases are warranted.

## Supplementary Information

**Additional file 1: Table S1.** Serum concentration of CTRP9 and APN in patients of different infarct stages.

## Data Availability

All data is ready to be uploaded on request.

## Abbreviations

ABC-ELISA: Avidin–Biotin Peroxdase Complex-enzyme-linked Immunosorbent Assay
APN: Adiponectin
CI: Confidence interval
CTRP: C1q/TNF-Related Protein
HDL: High-density lipoprotein
HMW: High-molecular weight
LAA: Large artery atherosclerotic
LDL: Low-density lipoprotein
LMW: Low-molecular weight
MMW: Middle-molecular weight
tAPN: Total adiponectin
