# Supplementary material for: Serum CTRP9 and high-molecular weight adiponectin are associated with ischemic stroke
Source: BMC Neurol. 2022 Nov 15;22:429. doi: 10.1186/s12883-022-02967-w (PMC9664773; doi:10.1186/s12883-022-02967-w)
Supplement: Supplementary file 1 — Additional file 1: Table S1. Serum concentration of CTRP9 and APN in patients of different infarct stages. [file 12883_2022_2967_MOESM1_ESM.docx]

# Table S1. Serum concentration of CTRP9 and APN in patients of different infarct stages

|  | **Control**  **(*n* = 182)** | **Stage Ⅰ**  **(*n* = 57)** | **Stage Ⅱ**  **(*n* = 54)** | **Stage Ⅲ**  **(*n* = 38)** | **Stage Ⅳ**  **(*n* = 115)** | ***P*-value** |
| --- | --- | --- | --- | --- | --- | --- |
| CTRP9(ng/ml) | 0.58±0.58 | 0.45±0.64 | 0.34±0.55**^a^** | 0.29±0.34**^ab^** | 0.49±0.68**^d^** | ＜0.05 |
| tAPN(μg/ml) | 13.69±8.50 | 12.08±9.62 | 10.63±8.24**^a^** | 9.83±6.59**^ab^** | 12.54±10.16**^d^** | ＜0.05 |
| HMW(μg/ml) | 13.13±8.54 | 11.54±9.89 | 10.00±8.18**^a^** | 9.08±6.23**^ab^** | 12.16±9.88**^d^** | ＜0.05 |
| MMW(ng/ml) | 7.27±5.96 | 12.34±17.44**^a^** | 6.70±7.88**^b^** | 5.83±6.36**^b^** | 6.12±5.55**^b^** | ＜0.001 |
| LMW(μg/ml) | 0.71±0.25 | 0.78±0.25**^a^** | 0.69±0.22**^b^** | 0.66±0.20**^b^** | 0.77±0.29 | ＜0.05 |

Notes: The measurements are presented as mean ± SD. 5 cases of undetermined onset date were not included in the staging sub-groups. a, statistically significant difference versus control; b, statistically significant difference versus stage I group; d, statistically significant difference versus stage III group.
